# Palbociclib sensitizes ER-positive breast cancer cells to fulvestrant by promoting the ubiquitin-mediated degradation of ER-α via SNHG17/Hippo-YAP axis

**DOI:** 10.1007/s10549-023-07138-0

**Published:** 2023-11-04

**Authors:** Lei Lei, Yuan Huang, Lei Shi, Weiwu Ye, Xianmei Lv, Lisha Ying, Xingfei Yu, Skye Hung-Chun Cheng, Yabing Zheng

**Affiliations:** 1 Department of Breast Medical Oncology, Zhejiang Cancer Hospital, Hangzhou, 310022 Zhejiang China; 2 Department of Radiation Oncology, Jinhua People’s Hospital, Jinhua, 321000 Zhejiang China; 3 Department of Breast Surgery, Zhejiang Cancer Hospital, Hangzhou, 310022 Zhejiang China; 4 Department of Radiation Oncology, Koo Foundation, Sun Yat-Sen Cancer Center, Taipei, Taiwan; 5 Zhejiang Cancer Institute, Zhejiang Cancer Hospital, Hangzhou, China

**Keywords:** ER-positive breast cancer, Fulvestrant resistance, Palbociclib, SNHG17, LATS1, ER-α

## Abstract

**Purpose:**

Endocrine therapy is the anti-tumor therapy for human breast cancer but endocrine resistance was a major burden. It has been reported that Palbociclib and fulvestrant can be used in combination for the treatment of patients who are experiencing endocrine resistance. However, the underlying mechanism is unclear. In this study, we aimed to investigate the mechanism by which Palbocicilib affected ER-positive breast cancer, combined with fulvestrant.

**Methods:**

We first detected the effect of palbociclib on cell survival, growth and cycle distribution separately by MTT, colony formation and flow cytometry. Then SNHG17 was screened as palbociclib-targeted LncRNA by LncRNA-seq, and the SNHG17-targeted mRNAs were selected by mRNA-seq for further determination. Subsequently, the underlying mechanism by which palbociclib promoted the cytotoxicity of fulvestrant was confirmed by qRT-PCR, western blot, and immunoprecipitation. Eventually, the xenograft model and immunohistochemistry experiments were used to validate the sensitization effect of palbociclib on fulvestrant and its mechanism in vivo.

**Results:**

Palbociclib significantly enhanced the cytotoxicity of fulvestrant in fulvestrant-resistant breast cancer cell lines. Interestingly, this might be related to the lncRNA SNHG17 and the Hippo signaling pathway. And our subsequent western blotting experiments confirmed that overexpressing SNHG17 induced the down-regulation of LATS1 and up-regulated YAP expression. Furthermore, we found that the increased sensitivity of breast cancer cells was closely associated with the LATS1-mediated degradation of ER-α. The following animal experiments also indicated that overexpressing SNHG17 obviously impaired the anti-cancer effect of co-treatment of palbociclib and fulvestrant accompanied by decreased LATS1 and increased ER-α levels.

**Conclusion:**

Palbociclib might sensitize the cytotoxicity of fulvestrant in ER-positive breast cancer cells by down-regulating SNHG17 expression, and then resulted in the LATS1-inactivated oncogene YAP and LATS1-mediated degradation of ER-α.

## Introduction

Breast cancer is one of the top main reasons for cancer-related mortality in females, and its incidence and mortality are also very high in China [1, 2]. Triple-negative breast cancer (TNBC) is immunohistochemically characterized by lacking gene amplified human epidermal growth factor receptor 2 (HER2), estrogen receptors (ER), and progesterone receptors (PR). Although TNBC is a highly malignant subtype of breast cancer, its’ proportion only accounts for approximately 15% to 20% [3, 4]. Others are the non-TNBC, for which the key therapeutic strategies are endocrine therapy [5–7]. Despite the rise in disease-free and overall survival (DFS, OS) of the initial phase of breast cancer patients in answer to these endocrine therapies, roughly 20% of patients taking these oral anti-estrogen medications develop endocrine resistance [8, 9]. Therefore, exploring novel therapeutic strategies to enhance the efficacy of endocrine therapies by conquering resistance in breast cancer is instantly required.

The development of cyclin-dependent kinase 4/6 (CDK4/6) inhibitors, including palbociclib, abemaciclib, and ribociclib, was a substantive breakthrough in the treatment of ER-positive, HER2-negative breast cancer [10, 11]. Palbociclib, approved by FDA in 2015, treated HR-positive, HER2-negative advanced breast cancer combined with an aromatase inhibitor (AI) as first-line treatment, or combined with fulvestrant in females who have undergone endocrine therapy (AI resistant-disease) [12]. However, the effectiveness based on real-world OS of palbociclib plus fulvestrant seems to be inferior to the efficacy published in clinical trials on advanced breast cancer [5]. More importantly, the underlying mechanism remains to be further elucidated. Thus, we need to find a more accurate mechanism for the combined therapy.

Long non-coding RNAs (lncRNAs) are transcripts at the lowest 200 nucleotides in length that have no or limited protein-coding capability [13, 14]. Following the previous studies, a mountain of evidence has certificated that the abnormal expression of lncRNAs is involved in the development and progression of breast cancer [15–17]. Moreover, lncRNAs are the key multifunctional molecules engaged in all kinds of malignant processes including tumorigenesis, metastasis, and progression by interacting with RNA, DNA, or proteins [18–21]. In this study, we confirmed that SNHG17 was the key lncRNA, whose suppression by palbociclib contributed to its’ sensitization functions on fulvestrant. Mechanically, palbociclib down-regulated SNHG17, and then induced LATS1-inactivated oncogene YAP and LATS1-mediated degradation of ER-α.

## Materials and methods

### Cells

The normal breast cancer cell lines T-47D and MCF-7 were purchased from ATCC and cultured in the medium of DMEM supplemented with penicillin (100 U/mL), streptomycin (100 μg/mL), and FBS (10%). The cells were cultured at 37 °C in a humidified atmosphere incubator with a 5% (v/v) CO_2_. All reagents for cell culture were obtained from Gibco (Carlsbad, CA, USA). The construction of fulvestrant-resistant breast cancer cell lines (hereafter, T-47D-F and MCF-7-F) was constructed referring to the published descriptions [22].

### MTT assay

Cell viabilities of T-47D-F and MCF-7-F were detected in vitro by using MTT assays. In brief, 5000 cells for both cell lines were seeded into each well of 96-well plates overnight to allow cell attachment. After that, the T-47D-F and MCF-7-F cells were treated with indicated concentrations of tamoxifen, exemestane, fulvestrant, abemacicilib, and palbociclib for 24 h. Then, 50 μl of 1 mg/ml MTT solution (Sigma-Aldrich, St. Louis, MO, USA, M2128) in PBS was added into each well and incubated at 37 °C for 3 h, and the purple crystal was dissolved by DMSO. Finally, the absorbance at 570 nm was read by a microplate reader (Molecular Devices, Sunnyvale, CA, USA).

### Colony formation assay

Briefly, the 300 T-47D-F and MCF-7-F cells were placed in a 6-well plate individually, and treated with indicated drugs. After 10 days of culture, the cells were discarded culture medium. After washing with PBS buffer twice, the colonies were fixed with methanol for 10 min. After that, the colonies were stained with crystal violet solution (aladdin, Shanghai, China, C110703). Finally, the colonies (> 50 cells) were numbered under a microscope.

### LncRNA and transcriptome sequencing

Human breast cancer cells MCF-7 were treated with Palbociclib or DMSO for 24 h, and three replications for each group were collected. Total RNA was extracted by Trizol reagent (Invitrogen, Carlsbad, CA, USA, 15596026) and subjected to LncRNA-seq by LC-Bio Technology CO. (Zhejiang, China). For transcriptome sequencing, MCF-7 cells were transfected with SNHG17 overexpression plasmid or empty vector for 24 h, and total RNA was extracted by Trizol reagent. Transcriptome sequencing and data analysis were also performed by LC-Bio Technology CO.

### Plasmid construction and transfection

The total sequence of lncRNA SNHG17 was synthesized and then subcloned into the vector pCDH to construct the SNHG17 overexpression plasmid. DNA sequencing was used to confirm the integrity of the plasmid construction. The Lipofectamine 2000 (Invitrogen, Waltham, MA, USA, 11668-019) was applied to perform the transfection according to the manufacturer’s instructions. Briefly, the plasmid and lipofectamine 2000 (Invitrogen) were mixed and the complex was formed after incubation for 20 min at RT. The complex was then added to a cell culture medium drop by drop and incubated for 24 h.

### Lentivirus infection

The SNHG17 stably expressed MCF-7-F cell line was constructed using lentivirus. Shortly, we purchased SNHG17-overexpressed lentivirus (Lenti-SNHG17) and corresponding control lentivirus (Lenti-control) from GeneChem (Shanghai, China), and then MCF-7-F cells were infected with Lenti-SNHG17 and Lenti-control (MOI = 200) overnight, respectively. Finally, the SNHG17 stably expressed MCF-7-F cells were screened using puromycin.

### Quantitative real-time PCR

Trizol (Invitrogen) was used to extract the total RNA from cell samples. The HiScript 1st Strand cDNA Synthesis Kit (Vazyme Biotech, Nanjing, China) was used to synthesize cDNA. Realtime PCR was performed on ABI-7500 using SYBR Green master mixture reagent (Thermofisher, Waltham, MA, USA, A46012). The PCR protocol was as follows: initial denaturation at 95 °C for 10 min, denaturation at 95 °C for 15 s for 40 cycles, and annealing and extension at 60 °C for 1 min. The 2^−ΔΔCt^ method was used to determine the relative expression of the target. β-actin is used as an internal reference gene. The primers were listed in the Table.1

**Table 1:** The sequence of primers

Name	F(5′-3′)	R(5′-3′)
β-actin	AGCGGGAAATCGTGCGTG	CAGGGTACATGGTGGTGCC
TYMSOS	GATTTCCAGGTCCCAGATG	CTTAGAGGCTCAACAACCC
SNHG17	AGTGGTATCCCGTGGTTC	GTGACGCTTCATGTGGTAG
TMPO-AS1	GGTTGGGCTATTGAGTTTGG	GAATGTAGACAAGAGGGATGG
TDRKH-AS1	GGACAGGAAGTCAAGGGAC	CCAGGGAGTTGGAGGTT
DLEU1	TCCAGCAGACTTCTACCC	CTCCCTTGAAAGACCACA
PPP1R26-AS1	TGACCATTCTGTCCTGTGGA	CTGAGTTGCACATCCCCTTC
DHRS4-AS1	ATCTACCTTCCGCCTGACTG	AGTGAAGGAACAGCACAGAC

### Western blotting

The T-47D-F and MCF-7-F cells were lysed in RIPA lysis buffer containing a 1% protease inhibitor cocktail (Sigma, St. Louis, MO, USA, P8215) for protein extraction. After electrophoresis on 10%, 12%, or 15% SDS–PAGE gels, proteins were electroblotted onto PVDF membranes (Millipore, Darmstadt, Germany, IPFL00010). After blocking with 5% non-fat milk for 2 h at room temperature, the membranes were then incubated with indicated primary antibodies at 4 °C overnight and secondary antibodies for 2 h at room temperature. After reacting with the HRP substrate, the chemiluminescence signals were visualized. The primary antibodies used in this study were as follows: YAP (CST, 14,074; dilution rate:1:1000), p-YAP (CST, 13,619; dilution rate:1:500), LATS1 (CST, 14,074; dilution rate:1:1000), p-LATS1 (CST, 9157; dilution rate:1:500), ER-α (CST, 8644; dilution rate: 1:1000), Ubiquitin (P4D1) (CST, 70,990, dilution rate:1:1000), and β-actin (Proteintech, 20,536–1-ap; dilution rate:1:5000).

### Immunoprecipitation assays

The SNHG17-overexpressed MCF-7-F cells for immunoprecipitation assays were firstly lysed by IP lysis buffer (Thermo Scientific, Massachusetts, USA, 87,787). Cell lysates (with 250–500 μg protein contained) were incubated at 4 °C with 1 μg anti-ER-α (CST, 8644; dilution rate: 1:50) and 4 μl flag-beads (Sigma, St. Louis, MO, USA, M8823) overnight. Then the protein-antibody-beats complexes was detected by WB experiments.

### Xenograft mouse model

Eighteen female Balb/c nude mice (4 weeks old, 20 g) were obtained from Vital River Laboratories (Beijing, China) to generate a xenograft mice model. Animal experiments were performed in compliance with the regulations and guidelines of institutional animal care of Zhejiang Cancer Hospital, and conducted according to the AAALAC and the IACUC guidelines. Xenografts were established by subcutaneously injecting 5 × 10^6^ SNHG17 stably expressed MCF-7-F cells into the right flank of nude mice. Tumor size was determined using a micrometer caliper. Tumor volume (mm^3^) was calculated using the following formula: *V* = (*a* × *b* × *c*)/2. After the largest tumors reached a volume of about 50–80 mm^3^, mice were randomly separated into three groups: (i) pCDH + control, (ii) pCDH + fulvestrant and palbociclib, and (iii) SNHG17 + fulvestrant and Palbociclib. 5 mg/kg fulvestrant was administered subcutaneously once per week, 25 mg/kg palbociclib was administered intraperitoneally daily, and all drugs treatment continued for 4 weeks. In this process, tumor size was measured every two days. After treatment, mice were euthanized and tumors were excised and weighed. The tumors were subjected to IHC experiments.

### IHC assays

For IHC experiments, 6 μM thick sections obtained from xenografts tissues were de-paraffinized and rehydration in graded ethanol. For antigen retrieval, slides were immersed in 0.01 M citrate buffer, pH 6.0, using a steamer at 95 °C. Subsequently, sections were incubated with primary antibodies (ki67, LATS1, and ER-α, diluted at 1:500 or 250 respectively in PBS) at 4 °C overnight. After washing with PBS, horseradish peroxidase-conjugated secondary antibodies were incubated. Slides were incubated with diaminobenzidene substrate for color development and counterstained with hematoxylin.

### Statistical analysis

All data are collected as the means ± S.D. from three independent experiments. All statistical analysis was determined by using GraphPad Prism 5.0 (GraphPad Software, Inc., La Jolla, CA, USA) and SPSS 13.0 (SPSS, Inc., Chicago, IL, USA) software packages. Statistical significance was performed using two-sided Student’s t-test for two groups and using one-way ANOVA for multiple groups. *P* < 0.05 (*) was considered statistically significant.

## Results

### Palbociclib enhanced the cytotoxicity of fulvestrant in T-47D-F and MCF-7-F cells

**Fig. 1:**
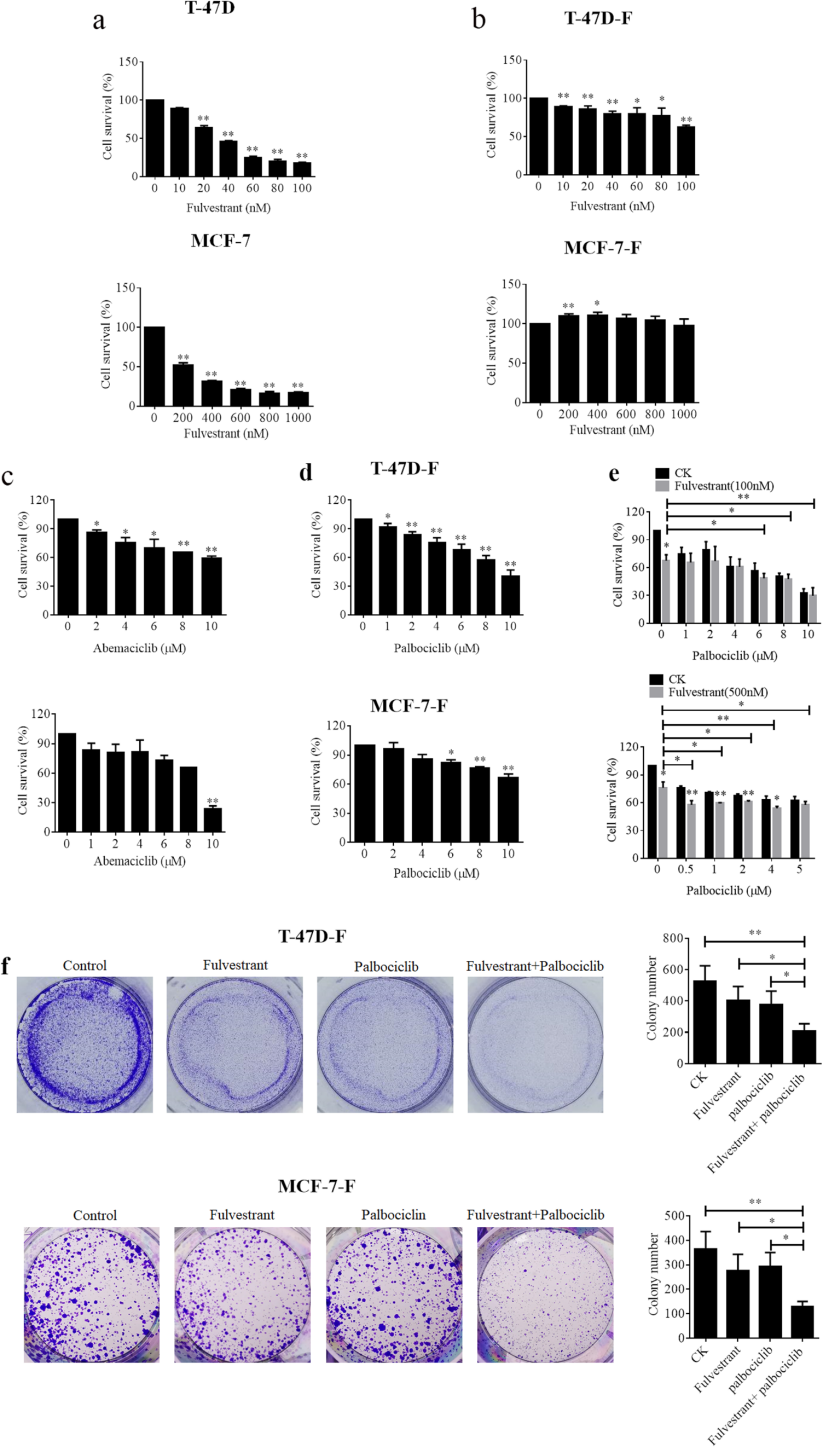
Palbociclib enhanced the cytotoxicity of fulvestrant in human breast cancer. **a**–**b** normal breast cancer cell lines (T-47D and MCF-7) and fulvestrant-resistant cell sublines (hereafter, T-47D-F and MCF-7-F) were incubated with the indicated concentrations of Fulvestrant for 24 h. Cell viability was determined by the MTT assay. **c**–**d** T-47D-F and MCF-7-F were incubated with the indicated concentrations of Abemaciclib (c), and Palbociclib (d) for 24 h. Cell viability was determined by the MTT assay. **e** The T-47D-F and MCF-7-F cells were treated with indicated concentrations of palbociclib alone and/or combined with indicated concentrations of fulvestrant for 24 h. Cell viability was determined by the MTT assay. **f** The T-47D-F and MCF-7-F cells treated with indicated concentrations of Palbociclib (1 μM for T-47D-F, and 0.5 μM for MCF-7-F) alone and/or combined with fulvestrant (100 nM for T-47D, and 500 nM for MCF-7) for 14 days. After that, colony formation was analyzed. Quantitative analysis of cell colonies was shown in the histogram. The data are derived from one of the three independent experiments. *P < 0.05 vs. control, **P < 0.01 vs. control

Fulvestrant, a selective ER down-regulator, is the typical endocrine therapy agent prescribed for patients with ER-positive breast cancer [23]. Here, we selected normal breast cancer cell lines (T-47D and MCF-7) and constructed fulvestrant-resistant cell sublines (hereafter, T-47D-F and MCF-7-F) to investigate whether the fulvestrant could inhibit human breast cancer cells or not. As shown in Fig. 1a, fulvestrant exhibited an apparent inhibitory effect on the cell survival of T-47D and MCF-7, while fulvestrant mildly suppressed cell survival even at a very high dose such as 1000 nM in MCF-7-F cells (Fig. 1b). Therefore, T-47D-F and MCF-7-F were used for the following exploration.

As CDK4/6 inhibitor is used as a combination agent for endocrine therapy, we chose abemacicilib and palbociclib to further confirm whether they could sensitize fulvestrant in ER-positive breast cancer cells. Firstly, we treated T-47D and MCF7 cells with various doses of CDK inhibitors abemacicilib and Palbociclib for 24 h, and MTT assay was used to check the cytotoxicity of two drugs. As shown in Fig. 1c and d, both abemacicilib and palbociclib caused a dose-dependent reduction in the cell viability in T-47D-F and MCF-7-F cells. Next, we also further confirm whether they could sensitize T-47D-F and MCF-7-F cells to fulvestrant. The data of the MTT assay suggested that co-treated with indicated concentrations of palbociclib can obviously strengthen fulvestrant cytotoxicity in T-47D-F and MCF-7-F cells (Fig. 1e), while no similar effect was observed in abemacicilib (data were not shown). Concurrently, as shown in Fig. 1f, co-treatment of palbociclib and fulvestrant apparently decreased efficiency to form colonies compared with control, palbociclib alone and fulvestrant alone groups in both T-47D and MCF-7 cells. The clonogenic survival fraction was numbered 206 ± 49 and 128 ± 22 for the combined group, 527 ± 97 and 364 ± 72 for the control, 401 ± 91, 276 ± 67 and 375 ± 88, 292 ± 58 for the fulvestrant alone, and palbociclib alone groups on T-47D and MCF-7 cells in the right histogram, respectively. Taken together, these results certified that palbociclib strengthened the sensitivity of ER-positive human breast cancer cells to fulvestrant.

### Palbociclib sensitized the cytotoxicity of fulvestrant in ER-positive human breast cancer cells by down-regulating lncRNA SNHG17

**Fig. 2:**
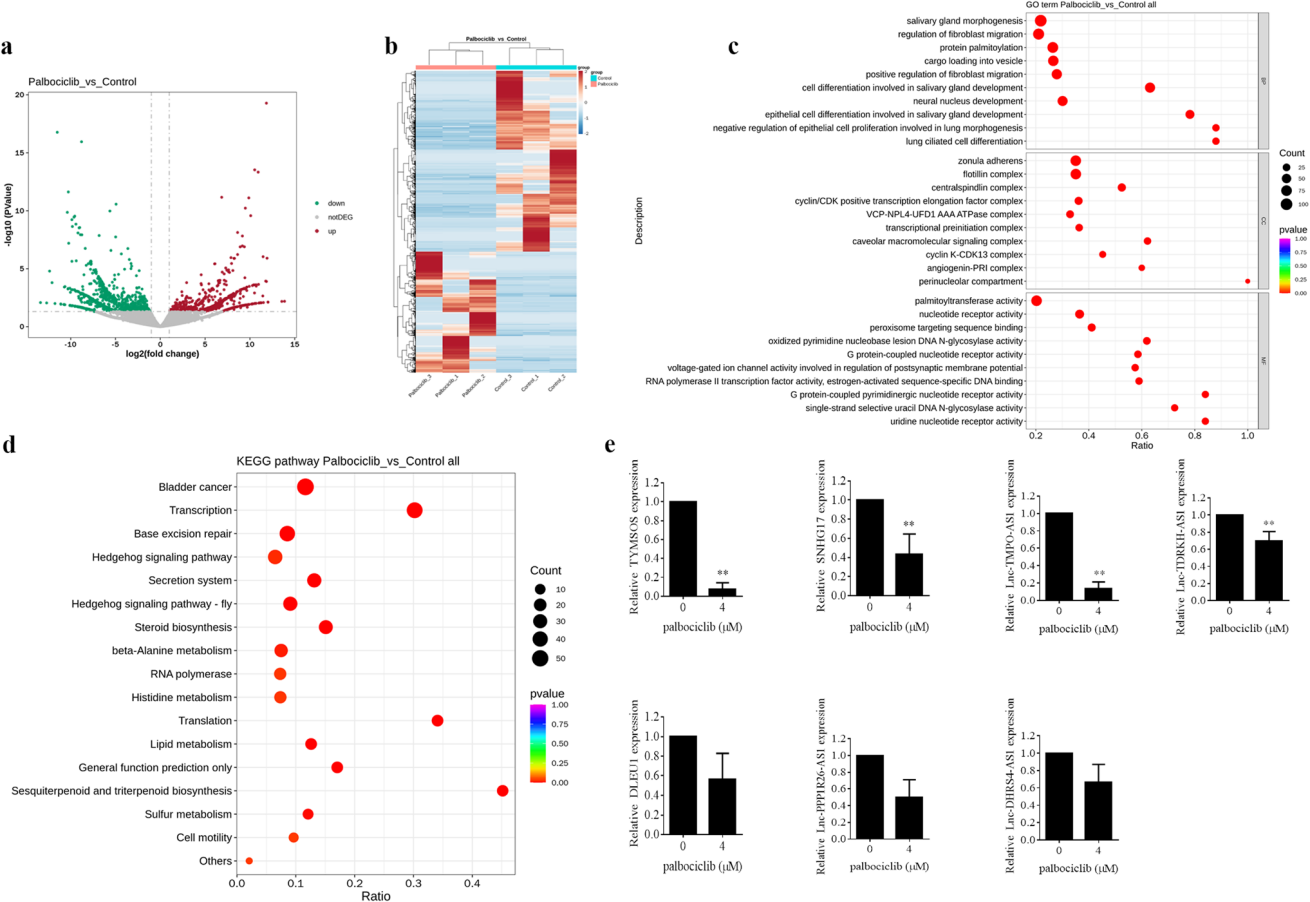
Palbociclib treatment resulted in the down-regulation of SNHG17. **a** Volcano plots showed lncRNAs expression in the Palbociclib compared to the control group in MCF-7-F cells. Red dots showed lncRNAs with significantly higher levels of expression, while the green dots represented lower ones. **b** The heatmap showed lncRNAs expression in the Palbociclib compared to the control group in MCF-7-F cells. The colors of the heatmap reflect log2-expression levels of lncRNAs in MCF-7-F cells. **c** Gene Ontology term enrichment analysis on the DEGs. **d** The KEGG pathway analysis on the DEGs. **e** The top 7 differentially expressed lncRNAs were subject to the following validation using qPCR. Data were presented by mean ± SD for three separate experiments. *P < 0.05 vs. control, **P < 0.01 vs. control

**Fig. 3:**
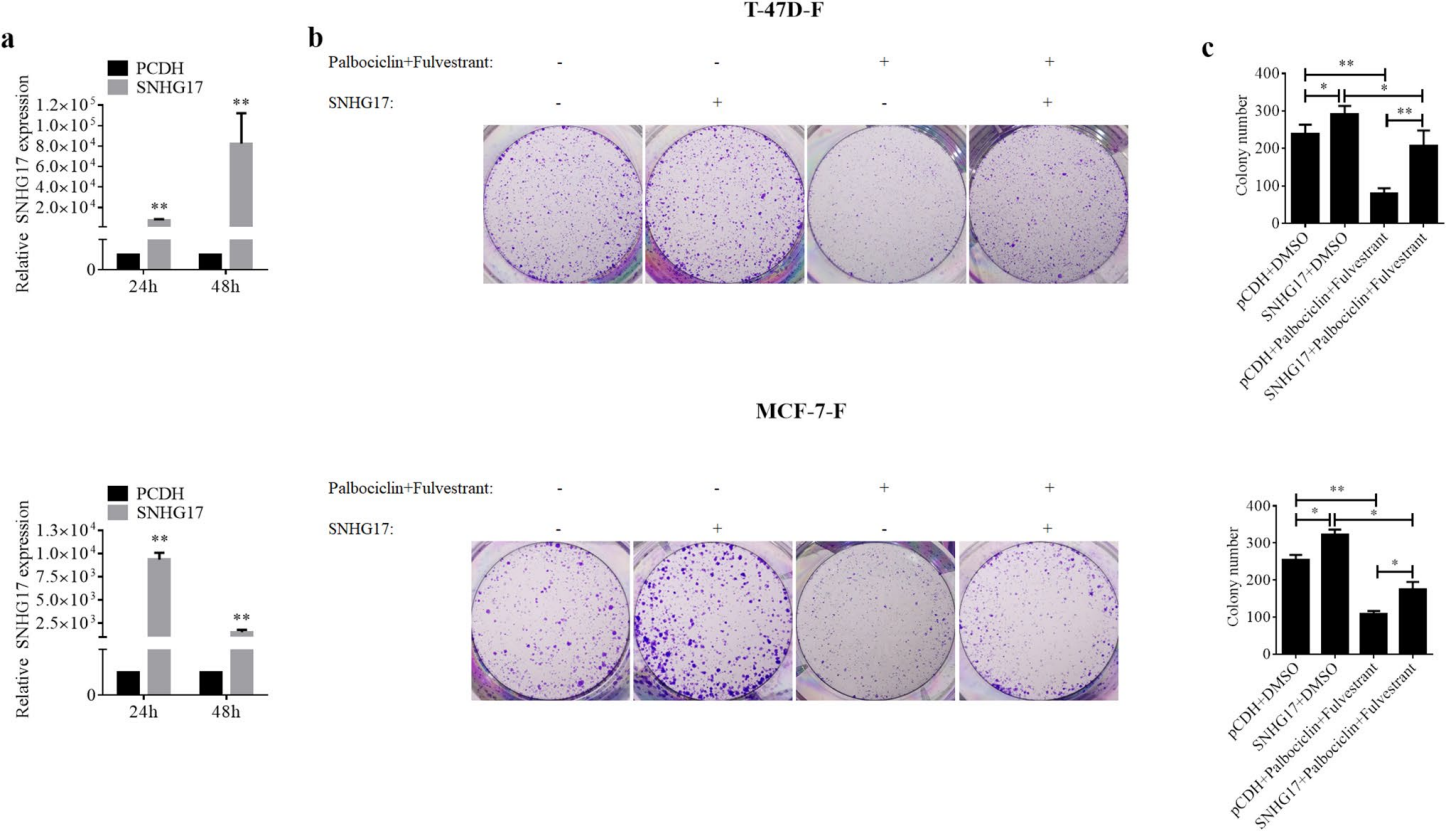
SNHG17 overexpression attenuated the role of Palbociclib on fulvestrant in ER-positive human breast cancer cells. **a** RT-PCR analysis detected the expression of SNHG17 in T-47D-F and MCF-7-F cells transfected with pCDH vector or SNHG17 overexpression plasmid. **b** The T-47D-F and MCF-7-F cells were transfected with pCDH or SNHG17 overexpression plasmid alone, or in combination with co-treatment with palbociclib and fulvestrant for 14 days. The doses for the two drugs were as same as in Fig. 1. After that, colony formation was analyzed. **c** Quantitative analysis of cell colonies was shown in the histogram. The data are derived from one of the three independent experiments. *P < 0.05 vs. control, **P < 0.01 vs. control

To identify the major mechanism involved in the sensitization function of Palbociclib on fulvestrant, high-throughput sequencing of LncRNA was performed in MCF-7-F cells treated with Palbociclib. At first, the RNA of MCF-7-F cells with palbociclib treated or untreated was isolated. Depending on the differentially expressed genes (DEGs) obtained from the sequencing, Volcano plots, and heatmap were generated and shown in Fig. 2a and b respectively. Furthermore, the enrichment analysis of Gene Ontology (GO, Fig. 2c) and Kyoto Encyclopedia of Genes and Genomes (KEGG, Fig. 2d) were performed in DEGs. We also chose the top 7 significantly differently expressed targeted lncRNAs for further identification in the palbociclib-treated MCF-7-F cells by RT-qPCR assay. As shown in Fig. 2e, palbociclib treatment remarkably reduced the expressions of lncRNA TYMSOS, SNHG17, TMPO-AS1, and TDRKH-AS1 in MCF-7-F cells. Although SNHG17 is not the most significantly suppressed lncRNA, it is reported that this lncRNA acts as an oncogene in multiple cancer types including breast cancer [24, 25]. We constructed the overexpression plasmid for SNHG17, and its transfection efficiency in both T-47D-F and MCF-7-F cells was verified by RT-PCR (Fig. 3a). The ectopic expression of SNHG17 alone obviously increased the colony formation in T-47D-F and MCF7-F cells in contrast to their control cells (Fig. 3b). More interestingly, SNHG17 overexpression also attenuated the synergistic effect of Palbociclib and fulvestrant in both two cell lines (Fig. 3b). The clonogenic survival fraction was numbered that 238 ± 25 and 255 ± 13 for the control, 291 ± 22 and 322 ± 14 for the SNHG17 alone groups, 80 ± 15 and 108 ± 8 for the fulvestrant and palbociclib combined groups, and 206 ± 42 and 175 ± 20 for the three combined groups in T-47D-F and MCF7-F cells in the right histogram, respectively (Fig. 3c). Taken together, these results suggested that Palbociclib enhanced the sensitivity of ER-positive breast cancer cells to fulvestrant by targeting SNHG17, at least partially.

### The LATS1-mediated YAP phosphorylation and ubiquitination of ER-α contributed to the sensitization effects of palbociclib on fulvestrant in ER-positive human breast cancer

**Fig. 4:**
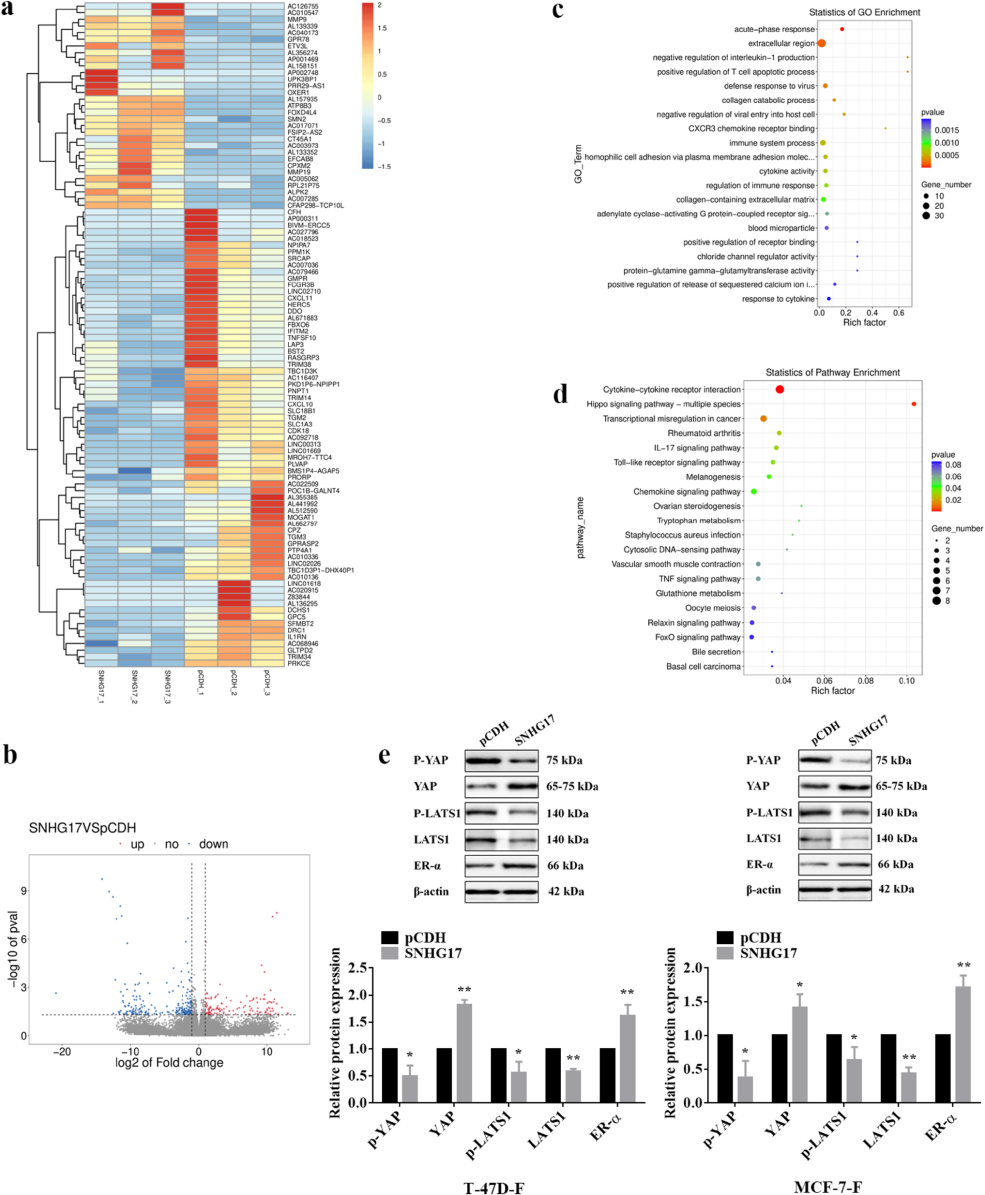
Overexpressing SNHG17 inhibited LATS1 expression and the phosphorylation of YAP. **a** The heatmap showed gene expression in the overexpression of SNHG17 compared to the control group in MCF-7-F cells. The colors of the heatmap reflect log2-expression levels of genes in MCF-7-F cells. **b** Volcano plots showed gene expression in the overexpression of SNHG17 compared to the control group in MCF-7-F cells. Red dots showed genes with significantly higher levels of expression while the green dots represented lower ones. **c** Gene Ontology term enrichment analysis on DEGs. **d** The KEGG pathway analysis on DEGs. **e** Western blotting assay was performed to determine the activation of Hippo-YAP signaling pathway and the expression of ER-α in T-47D-F and MCF-7-F cells after overexpressing SNHG17. Data were presented by mean ± SD for three separate experiments. *P < 0.05 vs. control, **P < 0.01 vs. control

To further elucidate the mechanism by which SNHG17 influences the sensitivity of fulvestrant in ER-positive breast cancer cells, mRNA sequencing was performed in MCF-7-F cells with SNHG17 overexpressed. According to the differentially expressed genes, the heatmap and Volcano plots were shown in Fig. 4a and b respectively. GO and KEGG pathway enrichment analyses were also performed, and the data from KEGG revealed that cytokine-cytokine receptor interaction, Hippo signaling pathway, and transcriptional misregulation in cancer were significantly enriched (Fig. 4c and d). It has been reported that the dysregulation of the Hippo-YAP signaling pathway has remarkable implications for the development of tumors, the activated large tumor suppressor 1 (LATS1) promoted the phosphorylation of YAP and then resulted in the degradation of YAP [26]. Thus, we next investigated whether the Hippo signaling pathway was activated under SNHG17 overexpression in ER-positive breast cancer cells. As shown in Fig. 4e, the inhibitory phosphorylation of YAP was inhibited and the YAP expression was up-regulated. Moreover, the expression of LATS1 and p-LATS1 were both suppressed, and the estrogen receptor-α (ER-α) expression level was significantly up-regulated.

**Fig. 5:**
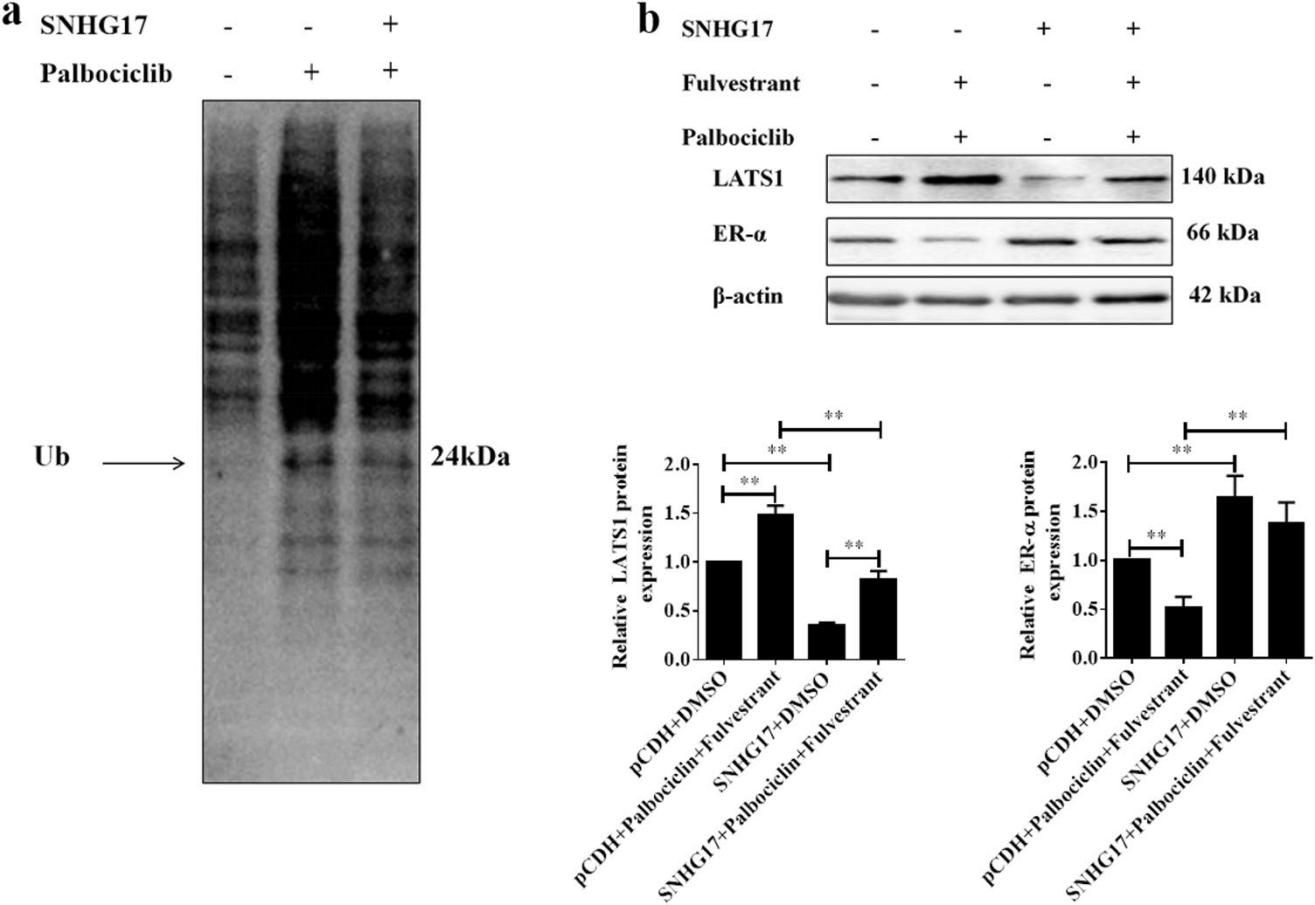
Palbociclib sensitized breast cancer cells to fulvestrant by promoting LATS1-mediated degradation of ER-α. **a** Immunoprecipitation assays and western blotting assays were combined to detect the binding between LATS1 and ER-α in SNHG17-overexpressed MCF-7-F cells, and then the expression of ER-α with ubiquitin was examined by western blotting in the immunoprecipitates. **b** The MCF-7-F cells were transfected with pCDH vector or SNHG17 overexpression plasmid followed by treated with DMSO, or co-treatment of fulvestrant and Palbociclib for 24 h, Proteins were extracted and subjected to Western blotting assay to assess of LATS1 and ER-α expressions. β-actin was used as a loading control. Quantitative analysis expression of proteins in the left showed in the right histogram. Data was presented by mean ± SD for three separate experiments. *P < 0.05; **P < 0.01

LATS1/LATS2 was reported to be related to the degradation of ER-α, and this effect is independent on their kinase activity or their downstream effectors YAP and TAZ. LATS induces the ubiquitin-mediated degradation of ER-α in a Ddb1–cullin4-associated-factor 1 (DCAF1)-dependent manner [27]. Therefore, MCF-7-F cells were transfected with LncRNA SNHG17 or not, and immunoprecipitation (IP) and western blotting (WB) assays were performed to explore the ubiquitination of ER-α. As shown in Fig. 5a, palbociclib treatment promoted the ubiquitination of ER-α, while overexpressing SNHG17 attenuated that result, indicating that the effect of palbociclib was closely related to the ubiquitin-mediated degradation of ER-α. For further validation, the MCF-7-F cells with LncRNA SNHG17 transfected or not were co-treated with or without palbociclib and fulvestrant. As shown in Fig. 5b, overexpressing SNHG17 not only notably down-regulated the protein level of LATS1 and up-regulated the protein level of ER-α in MCF-7-F cells but also recovered the promotional effect of the co-treatment of palbociclib and fulvestrant on LATS1 level and the inhibitory effect on ER-α level. Therefore, we could come to the conclusion that palbociclib might sensitize the cytotoxicity of fulvestrant to ER-positive breast cancer cells by inhibiting the expression of SNHG17, then increasing the expression of LATS1, and eventually, promoting the ubiquitin-mediated degradation of ER-α.

### SNHG17 overexpression effectively reversed the sensitization effects of Palbociclib on fulvestrant in MCF-7 cells derived xenograft

**Fig. 6:**
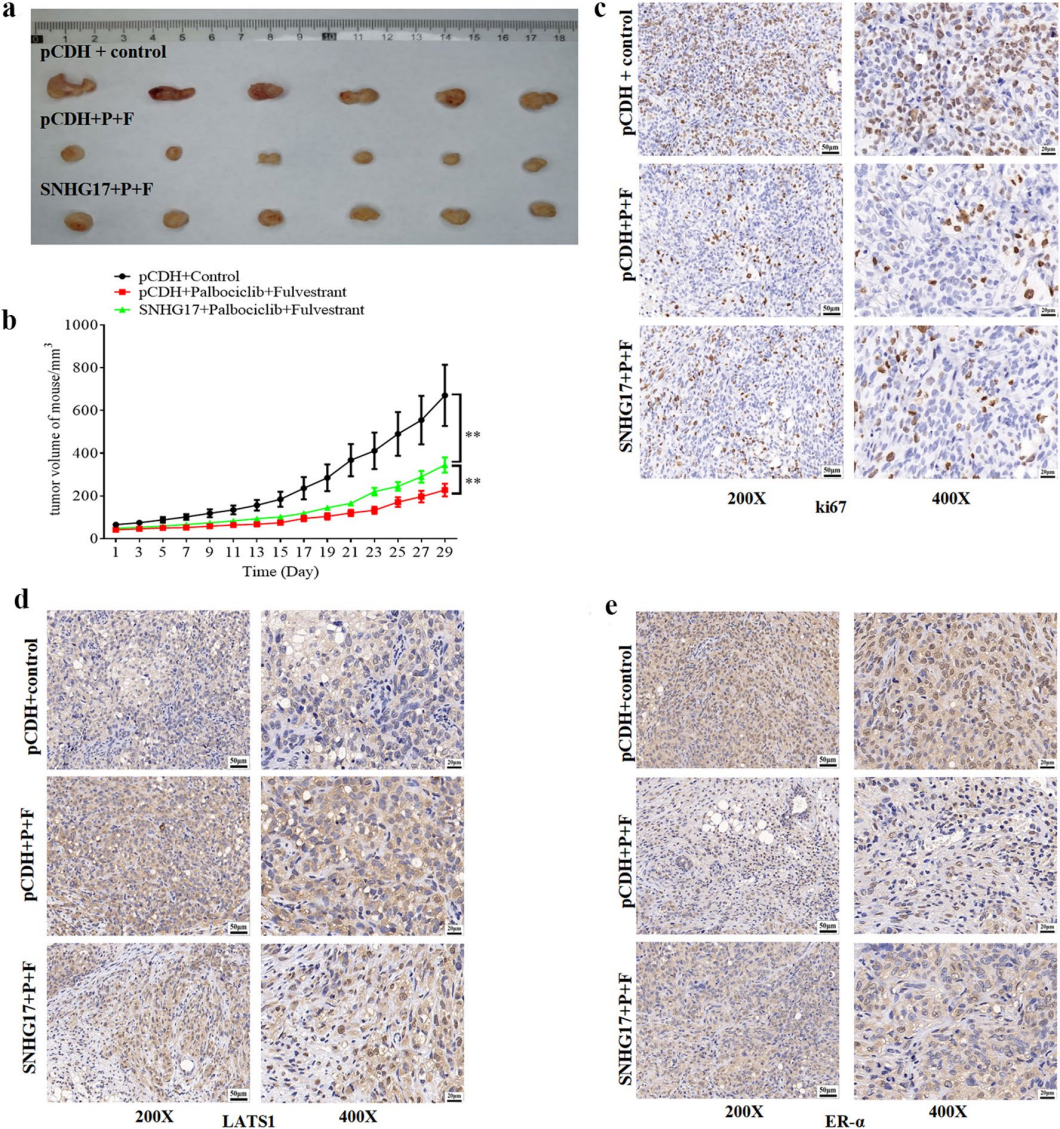
Overexpressing SNHG17 attenuated the sensitization effect of Palbociclib on fulvestrant in MCF-7 cells-derived xenografts by up-regulating ER-α via LATS1. **a** 5 × 10^6^ SNHG17 overexpressed or control MCF-7-F cells were subcutaneously injected into the right flank of nude mice to obtain xenografts. After the largest tumors reached a volume of about 50–80 mm^3^, mice were randomly separated into three groups: (i) pCDH + control, (ii) pCDH + fulvestrant and palbociclib, and (iii) SNHG17 + fulvestrant and palbociclib. N = 6 for each group. 5 mg/kg fulvestrant was administered subcutaneously once per week, 25 mg/kg palbociclib was administered intraperitoneally daily, and all drug treatment continued for 4 weeks. **b** Tumor size was determined using a micrometer caliper every two days. Tumor volume (mm^3^) was calculated using the following formula: *V* = (a × b × c)/2. **P < 0.01. **c**–**d** IHC assays were performed to detect the expression of ki67 (**c**), LATS1 (**d**) and ER-α (**e**) proteins. Representative data were shown. F: fulvestrant; P: Palbociclib

To further determine the role of SNHG17 in fulvestrant resistance in ER-positive breast cancer, SNHG17 stably overexpressed MCF-7-F cell line was constructed using lentivirus. After subcutaneously injected in nude mice, MCF-7-F cells-derived xenografts were obtained and followed by co-treatment of palbociclib and fulvestrant. Our data revealed that the combined administration of palbociclib and fulvestrant effectively suppressed tumor growth. However, overexpression of SNHG17 significantly attenuated the role of the two drugs (Fig. 6a and b). ki67 is a classic biomarker for cell proliferation, and we next used IHC assay to examine its expression in tumors. As shown in Fig. 6c, co-treatment with palbociclib and fulvestrant obviously inhibited the ki67 expression compared with these in the control, while SNHG17 effectively reversed this function. We also detected the expression of LATS1 and ER-α. As shown in Fig. 6d and e, palbociclib, and fulvestrant treatment markedly upregulated the LATS1 expression and downregulated the ER-α expression, while overexpression of SNHG17 reversed the above results.

## Discussion

ER-positive breast cancer is the most ordinary subclass of breast cancer, which is a therapeutic schedule that restrains estrogen production and/or targets the ER signaling pathway directly [8]. Although endocrine therapy has reasonably lessened recurrence and mortality in breast cancer, acquired resistance to this treatment still limits the therapeutic efficiency, and new therapeutic strategies to overcome this resistance are in urgent need. Fulvestrant, a selective estrogen receptor degrader of endocrine therapy drugs, is approved as the first- or second-line treatment for postmenopausal female with ER-positive breast cancer who has no response to tamoxifen [28–30]. Clinical studies have demonstrated that fulvestrant dramatically improved the progression-free survival of patients [6, 31–33]. However, fulvestrant develops resistance duration of therapy remains a clinical challenge. Therefore, it is of key importance to authenticate the factors and/or mechanisms related to fulvestrant resistance in breast cancer. In the present study, we selected two fulvestrant-resistant cell sublines (T-47D-F and MCF-7-F), and we confirmed that palbociclib significantly enhanced the cytotoxicity of fulvestrant in both two cell lines.

To identify whether lncRNA was involved in the sensitization effect of Palbociclib on fulvestrant, lncRNA sequencing was performed in MCF-7-F cells treated with Palbociclib. And, the result indicated that Palbociclib suppressed multiple lncRNAs including SNHG17, which functions as a pro-tumor lncRNA in multiple cancer types. Therefore, an overexpression plasmid for SNHG17 was constructed and allowed to transfected T-47D-F and MCF-7-F cells to verify whether SNHG17 was involved in the role of palbociclib on fulvestrant in ER-positive human breast cancer cells. The colony formation assay was performed and the data indicated that SNHG17 overexpression not only promoted the colony formation activities in two cell lines but also significantly reversed the role of palbociclib on fulvestrant. More importantly, similar results were also obtained in the nude mice xenograft models derived from MCF-7-F cells. Therefore, these data suggested that the down-regulation of SNHG17 contributed to the sensitization effect of palbociclib on fulvestrant in ER-positive breast cancer cells. However, little is known about how SNHG7 works in this procedure. Subsequently, mRNA sequencing assay was performed in MCF-7-F cells with SNHG17 overexpression. And the KEGG analysis revealed that the Hippo signaling pathway was enriched.

Mounting evidences indicate that the dysregulation of the Hippo signaling pathway is normal and is associated with the heterotypic expression of YAP/TAZ and the other genes [34]. Moreover, previous studies have certificated that YAP is associated with drug resistance, furnishing an attractive therapeutic target [35–37]. LATS1 and LATS2 (Hereafter, LATS1/2) are the hippo pathway kinases, and the inactivation (phosphorylation) of YAP/TAZ is regulated by activated LATS1/2, but activated YAP/TAZ regulated cell stemness and proliferation by translocated to nucleus [38]. This procedure represents the implementation of the main functions of the Hippo pathway. Interestingly, our results demonstrated that overexpressing SNHG17 significantly up-regulated the expression YAP and inhibited the phosphorylation of YAP, indicating that the palbociclib-promoted toxicity of fulvestrant in breast cancer may be related to the down-regulation of YAP, at least partially. Notably, it has been reported that LATS also mediates the interaction between Hippo and ER-α signaling. LATS can induce the ubiquitin-mediated degradation of ER-α through DCAF1 in a YAP/TAZ-independent manner [27]. Unsurprisingly, our further western blotting experiments confirmed that palbociclib significantly promoted the ubiquitination of ER-α but overexpressing SNHG17 abrogated that outcome. Moreover, the xenograft model also showed that overexpressing SNHG17 countervailed the inhibitory effect of palbociclib plus fulvestrant treatment on tumor growth, besides, the increased LATS1 level and decreased ER-α level induced by palbociclib plus fulvestrant treatment in xenograft tumor tissues were also reversed by overexpression of LATS1. Fulvestrant is regarded as an antagonist of ER, the increased sensitivity of fulvestrant to ER-positive breast cancer cells can be explained if palbociclib induces the LATS1-mediated degradation of ER-α via downregulating the SNHG17 expression.

## Conclusion

In conclusion, our study demonstrated that Palbociclib could sensitize ER-positive breast cancer cells to fulvestrant by inactivating the YAP and promoting LATS1-mediated degradation of ER-α via downregulating the SNHG17 expression. Therefore, these findings support that targeting SNHG17/LATS1 could supply a valuable therapeutic strategy to alleviate acquired endocrine resistance, especially fulvestrant resistance in ER-positive breast cancer.

## Data Availability

All data generated or analyzed during this study are included in this published article.
